# Analysis of Cross-Regional Transfer of Food Safety Risks and Its Influencing Factors—An Empirical Study of Five Provinces in East China

**DOI:** 10.3390/foods12081596

**Published:** 2023-04-09

**Authors:** Kai Li, Shijiu Yin, Yuanyan Chen

**Affiliations:** School of Economics, Qufu Normal University, Rizhao 276826, China

**Keywords:** cross-regional transfer of food safety risks, intelligent supervision, cross-regional cooperation, social network analysis, FE model with Driscoll–Kraay standard errors

## Abstract

The cross-regional transfer of food safety risks has become more prominent, bringing new challenges to food safety regulation. This study used a social network analysis to delve into the nuanced features and determinants of the cross-regional transfer of food safety risks based on the food safety inspection data of five provinces in East China from 2016 to 2020, thus contributing to the establishment of effective cross-regional cooperation in food safety regulation. The main findings are as follows: First, the cross-regional transfer of unqualified products accounts for 36.09% of all unqualified products. Second, the food safety risk transfer network presents a typical complex network—a relatively low but increasing network density, heterogeneous nodes, numerous subgroups, and a dynamic structure—bringing more difficulties to food safety cross-regional cooperation. Third, territorial regulation and intelligent supervision both contribute to restricting cross-regional transfers. However, the advantages of intelligent supervision have not yet been brought into play due to low data utilization. Fourth, the development of the food industry helps to mitigate the cross-regional transfer of food safety risks. To achieve effective cross-regional cooperation in food safety risks, it is essential to use food safety big data as a guide and to maintain synchronization between the development of the food industry and the improvement of regulations.

## 1. Introduction

Food safety has always been one of the most challenging social issues worldwide. A Chinese proverb states that “Food is the most essential necessity for the people, and food safety is of utmost importance”. Food safety concerns human health, well-being, and social stability. According to the estimation of WHO, 600 million people fall ill after eating contaminated food each year, resulting in 420,000 deaths and the loss of 33 million healthy life years. In China, the “China Modern Peaceful Development Index” survey conducted from 2012 to 2021 showed that, for ten consecutive years, food safety was the top concern among the top ten safety issues in China. The Chinese government has undertaken significant measures to reform the food safety regulatory system and to impose stricter regulations in response to widespread concerns about food safety issues. Despite these efforts, the pass rate for food safety inspections remains unstable, and consumers continue to lack confidence in food safety [1,2]. Therefore, it is still imperative to further improve the efficiency of food safety regulation.

However, because of the globalization of the food system, the food supply is becoming increasingly complex, and food safety risks are being increasingly shared across borders [3,4]. There is an urgent need to establish cross-regional and even cross-country cooperation in food safety regulation. In the context of globalization, the expansion of cross-border food trade has led to a deep integration and the reconstitution of global food production, transportation, and consumption. In the Internet era, the integration of the Internet and the food industry has given rise to various novel forms and models of food trade, expanding the scope of food trade across regions and even countries. As a result, the transfer of food safety risks has become increasingly cross-regional, posing a significant challenge to existing territorial food safety regulations [5,6]. According to the China Judicial Big Data Research Institute’s “Characteristics and Trends of Online Shopping Contract Disputes (2017.1–2020.6)” report, food disputes account for 45.65% of online shopping contract disputes, with 30.78% of these disputes involving food safety issues.

Characterizing food safety hazards and identifying critical control points serve as the bases for food safety regulation [7,8,9,10]. Since the melamine milk incident in 2008, the analysis of food safety risks has been a hot and unique spot in the field of food safety research in China. For example, based on 1460 food safety incidents collected from authoritative databases, such as the China Food Safety Resource Database and the National Food Safety Information Center from 2001 to 2010, Liu [11] established a food safety SC-RC matrix and found that food processing posed the most prominent risk in the food supply chain, with the improper use of food additives being the most significant problem. Similarly, Li et al. [12], Wen and Liu [13], and Zhu and Hong [14] reached a consensus conclusion based on food safety data from different sources for a similar period (2001–2012). Recent data confirm this conclusion, indicating that the sources of food safety risks and the vulnerabilities in risk regulation in China have not undergone fundamental changes. Zhang [15] analyzed 9314 food safety incidents exposed by the media from 2010 to 2019, confirming that the improper use of food additives remains the main cause of food safety problems and that food processing is still the riskiest stage. Tao et al. [16], based on the national food safety inspection data from 2017 to 2019, found that problems such as microbial contamination and the excessive use of additives were relatively prominent. In summary, there is a consensus on the sources and causes of food safety risks, with the food processing stage being the most significant area of concern due to the unregulated use of additives driven by economic interests.

As food safety risks caused by biological, chemical, and physical contamination can shift down the food supply chain and can be transferred between different regions or countries with food trade [17,18,19,20,21], it is necessary to clearly identify the weak components in the food supply chain and food safety regulation network. Some studies have therefore focused on the regional distribution of food safety risks. For instance, Li et al. [22] analyzed 2617 food safety incidents reported by a website from 2005 to 2014, and they found that food safety incidents were concentrated in economically developed and densely populated southern and eastern provinces in China. Similarly, Zhang [23] analyzed 6574 food safety incidents exposed by the media from 2007 to 2016, and they also found that food safety incidents were concentrated in eastern coastal provinces, such as Beijing, Guangdong, and Shandong. Yan et al. [24], based on 2131 pig meat safety incidents from 2009 to 2016, confirmed the existence of a significant cross-regional transfer feature in meat safety risks. Nie [25], based on 862,860 batches of inspection data extracted from the national and provincial market supervision and administration databases from 2017 to 2019, analyzed the spatial diffusion pattern of food safety risks and confirmed the existence of significant regional differences. The study also found that catering food and processed food were most likely to pose cross-regional risks [25].

However, the increasing complexity of the food trade network means that the transfer of food safety risks is not unidirectional and changeless [26]. Therefore, it is far from enough to find the weak link in the food supply chain or risky regions in the food safety regulation network at a certain point in time. We need to delve into more details of the food safety risk transfer networks in different regions and countries. We need to delve into more details of the critical nodes and specific paths of food safety risk transfer. Due to the availability of food safety risk data, existing research is limited to the food trade network and has not been extended to the food safety risk network [27,28,29]. In this paper, we used food safety inspection data from 2016 to 2020 and applied a social network analysis to establish a food safety risk transfer network of five provinces in East China. We explored the cross-regional transfer of food safety risks in these provinces and investigated the factors that affect such transfer, thereby providing policy implications for improving cross-regional cooperation on food safety supervision.

## 2. Materials and Methods

### 2.1. Method

A social network analysis utilizes the relationships between nodes as the fundamental unit of analysis to explore the structural variances in diverse social networks and their impacts on the network members. Granovetter’s analysis of the interdependent relationship between economic behavior and the social environment [30] has sparked a surge in the application of social network analyses in the field of economic management [31].

In this paper, we adopted 65 cities in the five provinces as network nodes and the transfer of unqualified food products from production cities to detection cities as network relationships to construct a food safety risk transfer network. East China is an important food production and sales area in China; it also plays a crucial role in food trade throughout the country [32]. We utilized food production and consumption characteristics as well as food safety regulation ability as primary screening criteria, while considering data availability and completeness. Eventually, five provinces, namely Shandong, Jiangsu, Anhui, Zhejiang, and Fujian, were chosen as the samples. Among these provinces, Shandong, Jiangsu, and Anhui had average meat, grain, and aquatic product outputs that exceed the average consumption, indicating that they are major food production regions (refer to Table 1). Meanwhile, Fujian had a significantly higher average output of aquatic products than consumption, with a slightly higher output of meat and a slightly lower output of grain, which makes it a major grain sales region. Zhejiang, on the other hand, had a higher output of aquatic products than consumption, but significantly lower outputs of meat and grain, making it a meat and grain sales region. The differences in food safety regulation among the sample provinces from 2016 to 2020 were also evident. Shandong and Zhejiang consistently received an A-level rating in the national food safety annual evaluation assessment during all five years, followed by Jiangsu with four times, and Fujian and Anhui with two times. In terms of the national food safety demonstration cities, Shandong had four cities, including Qingdao, Weifang, Yantai, and Weihai, while Zhejiang had two cities, Hangzhou and Ningbo. Jiangsu and Fujian had one city each, namely, Nanjing and Xiamen, respectively, whereas no city was selected in Anhui. Overall, the selected sample areas have a close food trade relationship, but they exhibit significant variations in food production and sales characteristics, as well as food safety regulation. Hence, these regions provide valuable insights for analyzing the transfer of food safety risks across regions and understanding the factors that influence such transfers.

The food safety risk transfer network *G* consists of node *V*, relationship *E*, and food safety risk transfer set *F*:

(1) $$G=\left(V,E,F\right)$$

where $V=\left(v_{1},v_{2},\ldots ,v_{n}\right)$ is a set of nodes, and $v_{n}$ represents 65 cities; *E* is a set of edges; each edge $e_{ij}$ has a pair of corresponding nodes $\left(v_{i},v_{j}\right)$ in *V*, representing the directed transfer relationship between the cities; $F=\left(f_{1},f_{2},\ldots ,f_{n}\right)$ is a set of food safety risk transfers, representing various types of unqualified food in the network. This network not only unveils the overall pattern and structural characteristics of food safety risk transfer across the 65 cities but also showcases the differences in the food safety risk transfer control abilities of various cities through variations in node attributes.

### 2.2. Data Collection

The main data used in this study were extracted from food safety inspection announcements published by the national and provincial Market Supervision and Administration Bureau between 2016 and 2020. We extracted food safety inspection information for 29 categories of food. Due to differences in the scope of inspections across the five years, we synthesized the various food production license regulations and annual food safety inspection plans of each province to establish classification criteria. After excluding information with unclear key information, such as the reasons for unqualified products, we obtained a total of 14,362 pieces of information. Considering that we needed to construct a food safety risk transfer network based on production address and sampled address, we further excluded information with unclear labeling of the production and inspection places, resulting in 12,234 pieces of valid information. Table 2 provides examples of both valid and invalid information.

This paper also utilized additional data, such as the selection status of a city and the year that it became a national food safety demonstration city, the launch date of open public data platforms, the growth of food-related industries (including agriculture, food, and catering), per capita GDP, urbanization level, and inclusive finance index, to analyze the factors impacting the transfer of food safety risks across regions in the sample provinces. The data sources for these indicators include the statistical yearbooks of the five provinces in East China and 65 cities, the Fudan University China Open Forest Index, and the Digital Inclusive Finance Index of Peking University, among others.

## 3. Results

### 3.1. Basic Statistics of the Cross-Regional Transfer of Food Safety Risks

**Figure 1:**
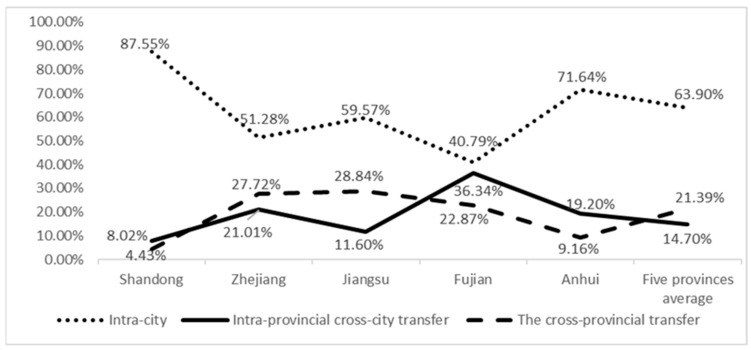
Average proportion of the cross-regional transfer of unqualified products in the sample provinces.

The cross-regional transfer of food safety risks has become a significant issue, as shown in Figure 1. The average proportion of the cross-regional transfer of unqualified products (produced in one city but detected in another city) in the five provinces is 36.09%, of which intra-provincial cross-city transfer accounts for 14.7%, and cross-provincial transfer accounts for 21.39%. However, the cross-regional transfer characteristics in each province varied significantly. Anhui and Shandong, being major food production regions, had the lowest proportion of cross-regional transfer, mainly intra-provincial cross-city transfer (with the proportion of cross-provincial transfer being 4.43% and 9.16%, and the proportion of intra-provincial cross-city transfer being 8.02% and 19.20%, respectively). However, Jiangsu, as a main food producing region, had a much higher proportion of cross-provincial transfer than intra-provincial cross-city transfer (28.84% and 11.6%, respectively), which was also higher than the main sales provinces, namely, Zhejiang and Fujian. Zhejiang and Fujian, being the main sales provinces, had more obvious characteristics of food safety risk transfer across regions (48.72% and 59.21%, respectively), but Zhejiang had a higher proportion of cross-provincial transfer, while Fujian had a higher proportion of intra-provincial cross-city transfer.

The top ten product categories with the most notable cross-regional transfer of risks were pastries, edible agricultural products, stir-fried and nut products, convenience foods, seasonings, etc., which, together, account for over 60% of the total (refer to Table 3). Pastries had the highest proportion of cross-regional transfer, with 663 batches of unqualified products detected (15.6% of the total), which was significantly higher than other product categories. Other products with a relatively high cross-regional transfer were also edible agricultural products (7.67%), stir-fried food and nut products (6.33%), convenience foods (5.32%), and seasonings (5.13%). Interestingly, this finding differed significantly from the high-risk categories identified in the national food safety inspection results from 2016 to 2020 (see Table 4). The fact that the product categories with a higher risk of cross-regional transfer were not consistent with the products with a lower overall inspection pass rate suggests that the current regulation and inspection approach aimed at identifying problematic products may not be effective in curbing the cross-regional transfer of food safety risks.

### 3.2. Analysis of Food Safety Risks Transfer Network

To effectively regulate the cross-regional transferred food safety risks, we have to understand the immanent structure of the food safety risk transfer network, obtain the dynamic trend of cross-regional food safety risk transfer by network density analysis, explore the weak points by network centrality analysis, and map connections between cities by network substructure analysis.

#### 3.2.1. Network Density

Network density is a metric that quantifies the degree of interconnectedness between nodes in a network. It is calculated by dividing the total number of actual connections in the network by the total number of possible connections. A higher network density indicates a higher frequency of the cross-regional transfer of food safety risks. This index can be used to gauge the extent of food safety risk transfer between the 65 cities in the five provinces, and it is expressed as follows:

(2) $$D=L/\left[N\times \left(N-1\right)\right]$$

where *D* is the network density, *L* is the number of actual connections in the network, and *N* × (*N* − 1) represents the maximum number of connections that the overall network could have.

As seen in Table 5, the overall trend of the food safety risk transfer network density in the five eastern provinces from 2016 to 2020 was increasing, indicating that food safety risks are becoming more frequently cross-regional. The binary network density increased from 0.0226 to 0.0822 in five years, a 363% increase. The weighted network density also generally increased during the same period. However, the absolute value of the network density remained relatively low, indicating limited connections between the 65 cities in the five provinces for the cross-regional transfer of food safety risks.

**Figure 2:**
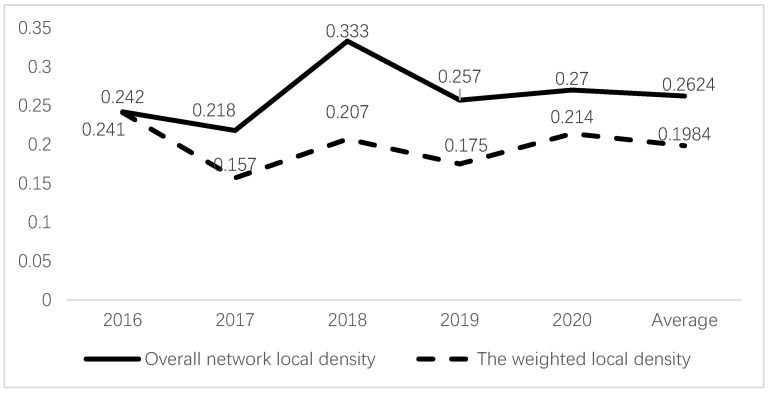
The local density of the food safety risk transfer network.

A further analysis of the clustering coefficient of each node (see Figure 2) revealed that the local density was much higher than the overall network density, indicating that there were specific cities with much tighter connections than other sample cities and suggesting the presence of subgroups in the food safety risk network of the five eastern provinces. The local density was calculated by first determining the connections of each city (i.e., the neighborhood), then calculating the local density, and finally obtaining the clustering degree of the entire social network by taking the average of all the city local densities. The overall network local density refers to the average of the local densities of all 65 cities, while the weighted local density was based on the local density and the scale of the subgroup network. Regardless of whether we consider the overall network local density or the weighted local density, the local density in each year was much higher than the overall network density shown in Table 5.

#### 3.2.2. Network Centrality

Network centrality is a measure of the number of direct or indirect connections that a node has in the network, reflecting its power and central position in the network. A high network centrality indicates that a city occupies a central position in the food safety risk transfer network, which implies that the city is a key node in food trade and the transfer of food safety risk. However, it also suggests that the city’s regulatory ability to curb the cross-regional transfer of food safety risk is weaker.

This paper examines the centrality of the food safety risk transfer network by analyzing the overall network centralization and node degree centrality. The overall network centralization captures the variation in the positions of all nodes in a network and is represented by the ratio of the network to a perfectly star-shaped network of the same size (i.e., the most centralized or unequal network). However, degree centrality is defined as the number of direct connections that a node has with other nodes in the network. The higher the degree centrality, the more transfer-in or transfer-out of food safety risk the city has and the higher its centrality in the network.

In terms of the overall centralization of the network (refer to Table 6), the mean centralization was only 7.47% from 2016 to 2020, indicating a low concentration of the network and suggesting that there are many paths for food safety risk transfer among the 65 cities in the five eastern provinces and no dominant central city. Regarding degree centrality, the average degree centrality of the 65 cities was 14.516, showing a trend of rising and then declining, with 2018 as the dividing year, indicating that the regulation of the cross-regional transfer of food safety risks was strengthened after 2018. The difference in centrality among the different cities can be seen from the maximum and minimum values of centrality. The maximum centrality reached 356 in 2018 and 285 in 2019, while the minimum was 1. At the same time, there was also a significant difference in the centrality of the same city in different years. For example, the degree centrality of Hangzhou was 19, 21, 41, 54, and 46 from 2016 to 2020, indicating a significant difference.

#### 3.2.3. Network Substructure

The substructure and internal groups of the food safety risk transfer network were analyzed using two criteria: cliques and N-Clan. A clique is defined as a group of nodes that are all directly connected to each other, with each city having direct connections with all other cities in the subgroup. In contrast, the N-Clan (with a set path length of 2) relaxes this restriction by allowing one city to connect with other cities in the subgroup through its directly connected nodes (but it requires that the cross-distance or path length between all cities in the subgroup does not exceed 2).

Based on the analysis results presented in Table 7, the food safety risk transfer network showed a high degree of decentralization, with 407 cliques and 21 N-Clan subgroups identified in the total network merged from 5 years of data from 2016 to 2020. The number of subgroups increased over time, indicating that the differentiation of subgroups is intensifying. The existence of subgroups means that cooperation strategies may need to be tailored to different groups, but if the number of subgroups continues to increase and become too many, then it may indicate that the food safety risk transfer path is more unstable, posing challenges for regional cooperation in food safety regulation.

### 3.3. Influencing Factors of the Cross-Regional Transfer of Food Safety Risks

#### 3.3.1. Model Setting

Explained Variable. This paper used the degree centrality of each city in the food safety risk transfer network as the dependent variable to measure the performance of each city in food safety risk across regional transfer regulation. Compared to the use of unqualified products or the pass rates of inspection as variables in previous studies, degree centrality has two unique advantages. First, it takes into account both the transfer-in and transfer-out of unqualified products, providing a more accurate reflection of a city’s regulatory efficiency in terms of food safety risk. Second, the degree centrality also considers the spatial relationships between a city’s food trade and food safety risk transfer with other cities, which is important in determining the key points and cooperative partners of food safety risk governance within specific spatial scopes.

Due to the relatively low network density and numerous subgroups within the food safety risk transfer network in the five eastern provinces, it is more appropriate to use the degree centrality within subgroups rather than the entire network. The data showed that only a small proportion of all cross-provincial transfers of unqualified products occurred between the 65 cities in the five provinces, and the use of the entire network may lead to an overestimation of the regulatory performance of cities with no food trade with other cities. Therefore, this paper chose to calculate the degree centrality of each city within each province to reflect the difference in the node’s position in the food trade and the food safety risk transfer within the subgroup. To avoid the impact of the scale difference of the five provincial subnetworks, this paper adopted standardized degree centrality.

Explanatory variables. The core explanatory variables for this study were the food safety regulatory capacity and industrial development level, selected based on the core contradiction between the “big food industry” and “weak food safety regulation” and the relevant literature on food safety regulation both domestically and abroad [33,34,35,36,37]. Additionally, control variables, such as per capita GDP, the Engel coefficient, the urbanization rate, and the level of digital financial payment, were also selected (see Table 8).

The territorial food safety regulatory capability was indicated by whether a city was selected as a national food safety demonstration city. The evaluation of the national food safety demonstration city was based on five aspects: basic work (such as joint responsibilities of the party and government, work mechanism, regulations, source control, and social governance), capacity (such as investment protection, equipment, inspection, and testing), production and operation status (such as enterprise management responsibility, process control, and product traceability), food safety situation (including public satisfaction, awareness, and creation rate, and the pass rate of random inspections), and demonstration leadership (such as credit supervision and mechanism innovation), which can measure the differences in the territorial food safety regulatory capability of cities more systematically and comprehensively. During the sample period, the country launched three rounds of national food safety demonstration cities in 2014, 2015, and 2016. If a city was selected in this period, it was recorded as 1; otherwise, it was recorded as 0.

The intelligent supervision capability was indicated by the launch time of each city’s data open platform. With the continuous integration of the Internet and the food industry, the Internet has, to some extent, reshaped food safety regulation and promoted the development of food safety intelligent supervision. As food safety intelligent supervision is still in the data-driven stage [38], the openness of food safety-related data is not only a crucial part of digital platform construction but also an important prerequisite for achieving the cross-regional collaborative governance of food safety. Therefore, the launch time of the data open platform was selected to measure differences in intelligent supervision capability.

The development of the food industry was measured by its industrial scale. A fragmented and scattered food industry is an important factor affecting the efficiency of food safety regulation [39]. Improving the scale level of the food industry not only helps to reduce regulatory pressure but also enhances industry technology and management capabilities through the economies of scale, thereby enhancing self-regulation within the industry. Therefore, this article selected the total value of agriculture, the total value of food industry (including the processing of agricultural products; food manufacturing; and the manufacture of wine, beverages, and refined tea), and the operating revenue of catering and accommodation above the quota as representative indicators of the development scale of agriculture, food industry, and catering, respectively. Given the significant differences in industrial scale among cities, logarithmic processing was adopted in the measurement analysis.

Based on the above variables, considering that the sample data belong to a typical “short panel”, the food safety risk transfer impact factor analysis model was set as follows:

(3) $$Degree_{\_}{}_{it}=\alpha +\beta x_{\_}{}_{it}+\mu_{\_i}+\varepsilon_{\_it}$$

In the equation, $degree_{\_}{}_{it}$ represents the centrality of city *i* in period *t*; $x_{\_}{}_{it}$ are various factors that affect the transfer of food safety risks across regions, including food safety supervision capacity and industrial development level as two core explanatory variables, as well as per capita GDP, the Engel coefficient, and other control variables, all of which change over time; $\mu_{\_i}$ represents the individual effect; and $\varepsilon_{\_it}$ is a random disturbance term.

#### 3.3.2. Model Test

The first step was to conduct an individual effects test (see the individual effects test in Table 9) to determine if there was heterogeneity in the cross-regional transfer of food safety risk among the different cities. The results showed that F (64, 251) = 2.11, Prob > F = 0.0000, which rejects the hypothesis that there are no individual effects at the 1% level. Thus, compared to mixed models, it was more appropriate to choose a fixed effects or random effects model.

Next, the Hausman test was performed to determine whether to choose a fixed effects model or a random effects model. If the individual effects are related to the explanatory variables, a fixed effects model should be selected. Otherwise, a random effects model should be chosen. The result of the Hausman test (see “Hausman test” in Table 9) at the 1% level rejected the null hypothesis (Prob > chi2 = 0.0000), indicating that the fixed effects model was better.

After selecting the fixed effects model, further cross-sectional autocorrelation tests and heteroscedasticity tests were conducted (see Table 9). The cross-sectional autocorrelation test indicated that there was a cross-sectional correlation problem, the sequence autocorrelation test result showed that there was sequence autocorrelation, and the heteroscedasticity test result showed that the model had a significant heteroscedasticity problem. Therefore, to correct these issues, the Driscoll–Kraay standard error model was chosen as the final model.

#### 3.3.3. Empirical Results

Based on the empirical results presented in Table 10, it is evident that there exists a significant negative correlation between national food safety demonstration cities and the degree centrality. This implies that enhancing the territorial regulatory capacity can effectively limit the cross-regional transfer of food safety risks. To tackle regulatory conflicts under the multi-departmental regulatory model, the 2018 food safety regulatory reform prioritized improving territorial regulatory capabilities and strengthening the comprehensiveness of regulation [40]. Demonstration cities emphasize the regulation of high-risk stages and key food market entities, enabling them to have greater food safety transfer-in and transfer-out regulatory capabilities. They also emphasize production and operation entities’ accountability and mass participation, laying the foundation for improving regulatory efficiency through social co-governance. This conclusion highlights the exemplary role of national food safety demonstration cities. Furthermore, in the absence of fixed pathways for the transfer of food safety risks in the sample areas, it is difficult to identify stable partners and governance objects for cross-regional cooperation governance. As a result, the importance of the territorial regulatory capacity is greatly enhanced.

Although the impact of open data platforms on regulating the cross-regional transfer of food safety risks was negative, it was not significant. This indicates that the openness of food safety data has not been effective in regulating such risks, although it should serve as the foundation for promoting cross-regional cooperative governance. Currently, the focus of digital government construction and food safety intelligent supervision is on the online transfer of traditional services and regulatory operations [41], with heavy reliance on digital technology [42]. However, true intelligent supervision, which promotes multi-party, multi-department, and multi-tool coordination for precise and efficient governance [43,44], is still a long way off. Due to the limited openness and utilization of food safety data, the risk warning function of food safety big data has not been realized. As a result, there is a lack of horizontal collaboration between governments and multi-party collaboration between government departments and other stakeholders, exposing the current shortcomings of intelligent supervision in dealing with the cross-regional transfer of food safety risks.

Agricultural development has a significant inhibitory effect on the cross-regional transfer of food safety risks, fully demonstrating the importance of industrial production and operation entities in food safety risk regulation. The larger the scale of the agriculture, the more prominent the restrictive effect on the cross-regional transfer of food safety risks (significant at a 10% level). Quality and safety are the lifeline of agriculture, and the larger the scale of the industry, the more prominent the importance of food safety for the industry. This leads to higher levels of attention and stronger food safety supervision by industry and regulatory entities, ultimately reducing the probability of food safety risks being transferred out [45]. Moreover, the improvement of the industry scale is often accompanied by the upgrading of industrial technology and the optimization of the industrial-organizational structure [46,47,48], and the improvement of technological innovation and industrial scale also effectively improves the overall food safety supervision capacity of the industry, thus reducing the possibility of food safety risk transfer-out [37,38]. In addition, regardless of the improvement of industry scale or quality improvement, they help to raise the territorial consumers’ attention to food safety and better exert the supervision role of consumers, thus reducing the possibility of food safety risk transfer-in. The development of large-scale catering also helps to inhibit the cross-regional transfer of food safety risks, but the effect is not significant. The development of the food industry has exacerbated the cross-regional transfer of food safety risks. This is because the development of the food industry is often accompanied by the elongation of the supply chain and the expansion of product markets, which have higher requirements for the cross-regional collaborative governance of food safety, regardless of the former or the latter.

From a control variable perspective, the urbanization rate has a significant positive impact on the cross-regional transfer of food safety risk, which is consistent with Zhang et al. [49]. This is primarily due to the fact that higher urbanization rates correspond to a lower self-sufficiency of urban agricultural products and food, thereby increasing dependence on external food sources [50]. Additionally, industrial and environmental pollution resulting from the urbanization process can further exacerbate food safety risks [51], leading to a higher likelihood of cross-regional contamination. The impact of the digital financial payment index is also significant, indicating that the development of digital payments, to some extent, promotes the cross-regional transfer of food safety risks. This is mainly because digital payments are closely related to the development of e-commerce, which is characterized by a higher spatial separation, virtual transaction parties, and concealed transactions [52,53]. These factors make regulation more challenging, particularly in the absence of effective regulatory frameworks for e-commerce, which has become an important channel for the cross-regional transfer of food safety risks.

#### 3.3.4. Robust Test

Due to data availability limitations, it is challenging to find additional territorial regulatory and intelligent governance variables that could be used to replace the core variables. Consequently, this paper employed the same model with censored data to conduct robust testing. By truncating 1% of the explained variables, industrial development, and control variables, we were able to minimize the impact of abnormal values on the regression results. As a result of truncating multiple variables, we removed 57 samples from the analysis. The regression results showed that the impact of the territorial regulatory capacity and intelligent governance on the cross-regional transfer of food safety risks is consistent with the original regression results (see Table 11). Specifically, the improvement of the territorial regulatory capacity and governance helps to curb the cross-regional transfer of food safety risks, but only the former is statistically significant, thus confirming the robustness of the regression results. The impact of agricultural development on the cross-regional transfer of food safety risks is significantly negative, which aligns with previous research. Regarding control variables, the impact of digital financial payment is also robust, while the impact of the urbanization rate is significantly reduced.

## 4. Discussion

Previous studies reached the consensus that, under the impetus of food trade, the global food network and food system are becoming increasingly complex, which poses challenges to food safety regulation. In contrast to the existing studies that focus on the analysis of food trade networks or the static analysis of regional differences in food safety risks distribution, one main purpose of this paper was to delve into the nuanced features and determinants of food safety risk cross-regional transmission in China, thereby enhancing cross-regional collaboration in the supervision of food safety.

The research results confirmed the theoretical analysis that food safety risks are increasingly shared across borders. The statistical analysis indicates that the cross-regional transfer of food safety risks can no longer be ignored. From 2016 to 2020, the cross-regional transfer of unqualified products in the five provinces in East China accounted for 36.09% of all unqualified products. Additionally, this study found that the food safety risk transfer network of the five provinces in East China presents a typical complex network. First, there are many nodes, but the network density is low, indicating that the transfer of food safety risks is limited to specific regions. Second, there is obvious heterogeneity among the nodes, and each node’s degree centrality varies significantly between different years, suggesting differences in their performance to control food safety risk transfer. Third, the network has numerous subgroups, which increased in number, indicating that the cross-regional transfer of food safety risks is constantly evolving due to the instability of cross-regional cooperation partners.

The study also confirmed the impact of the cross-regional transfer of food safety risk on the existing food safety regulatory system, highlighting the importance of cross-regional collaboration in food safety regulation. The issue of the cross-regional transfer of food safety risks is increasingly pressing, and there is no fixed path for such transfer, emphasizing the significance of cross-regional cooperation in food safety governance, as well as the challenges involved in such governance. Both the regulation and the development of food-related industries help to mitigate the cross-regional transfer of food safety risks, which is in line with previous research on food safety regulation. The conflict between the “diverse, small, dispersed” food industry and the limited regulatory resources is believed to be an impediment to improving China’s food safety regulatory efficiency. Therefore, inter-regional risk collaboration must prioritize the synchronization of industrial development and regulatory improvement. However, due to low data utilization, namely open data and the online transfer of traditional services and regulatory operations, the advantages of intelligent supervision have not yet been brought into play. To unlock the advantages of intelligent supervision, there is a need to deepen “Internet + supervision”, promote the transformation of intelligent regulation from leveraging Internet technology to collaborating with it and rebuilding the regulatory process, and improve the participation of industry bodies and consumers.

The study also has some limitations. First, due to the limited coverage of the traceability system, the problem of incomplete information in some food categories is more serious. For example, it is hard to record the production information of agricultural products, which may lead to a possible underestimation of the degree centrality of the main cities and provinces producing agricultural products. Second, due to the lack of food trade data at the regional level, in this study, we did not compare the food trade network with the food safety risk transfer network directly, which may provide more information about the food safety regulatory capacity differences among regions. Overall, while the study has some limitations, it provides valuable insights into the food safety risk transfer network in China and highlights areas for further research.

## 5. Conclusions and Policy Implications

### 5.1. Conclusions

This article took five provinces in East China as an example, based on food safety inspection data from 2016 to 2020, to analyze the basic characteristics and influencing factors of the cross-regional transfer of food safety risks in China.

The study drew several main conclusions. First, the integration of the Internet and the food industry has led to changes in the production and sales modes of the food industry, making the cross-regional transfer of food safety risks a significant concern. From 2016 to 2020, the cross-regional transfer of unqualified products in the five provinces in East China accounted for 36.09% of all unqualified products. The most significant risk spillover occurred for pastries, edible agricultural products, roasted and nut products, convenience food, condiments, etc. Second, the food safety risk transfer network in five provinces in East China presents typical complex network characteristics—a low density, heterogenetic nodes, numerous subgroups, and dynamic changes. While the increasing network density indicates a rise in the frequency of food safety risk transfer between the provinces, the low centrality and dispersed subgroups highlight the potential instability in cooperation partners for inter-regional cooperation in food safety risk regulation. Third, territorial food safety regulation and intelligent governance both help to restrict the cross-regional transfer of food safety risks, but only the former is significant; limited by the level of data utilization, the advantages of intelligent governance have not yet been fully utilized. Fourth, agricultural development has a significant inhibitory effect on the cross-regional transfer of food safety risks, emphasizing the importance of the self-management of market entities as the first responsible party in food safety.

### 5.2. Managerial Contributions

The main contributions of this study are trifold. First, the existing static analyses—of the sources, causes, and regional differences of food safety risks—are inadequate to resolve the conflicts between territorial regulation and inter-regional or even national food safety risk transfer. By comparing production and inspection places, the dynamic cross-regional transfer processes, key nodes, and specific paths of food safety risk were clearly identified. Second, social network analysis methods were introduced into the analysis of food safety risks for the first time. Food trade between regions is characterized as “many points, broad area, long chain, and dynamic”, which results in the food safety risk transfer network presenting typical social network characteristics: many nodes and obvious heterogeneity between nodes, a complex network structure, and dynamic changes. Therefore, it could not be more appropriate to use social network analysis methods to detail the structure of the food safety risk transfer network and to clarify the transfer characteristics of food safety risks between different cities. Third, in addition to the traditional influencing factors, such as territorial regulation and industrial development, this paper took an analytical look at the potential role of intelligent supervision in contributing to the cross-regional cooperative governance of food safety risks.

### 5.3. Policy Implications

To achieve an effective governance of food safety risks, it is essential to use food safety big data as a guide and to maintain synchronization between the development of the food industry and the improvement of regulations. The government must promote the transformation of food safety regulation from leveraging Internet technology to collaborating with it and rebuilding the regulatory process. By installing sensors in the production and circulation links of food, deeply analyzing inspection data, and training algorithms, the government can accurately describe the characteristics of cross-regional food safety risk transfer, identify abnormal food production and circulation situations, and take preventive measures in advance. Through a nationwide food traceability system based on blockchain technology, the government can realize full-process traceability from production to consumption and can improve the effectiveness of food safety regulation. Additionally, the government should promote the restructuring of regulatory processes. This will optimize the coordination of food safety regulatory resources based on overall risk, establish a cross-regional cooperation mechanism, and enhance the regulatory efficiency of food safety risks across regions. Furthermore, the government should leverage Internet technology to achieve multi-party governance. While strengthening regulatory capacity building and promoting food safety intelligent governance, the government should also promote open digital platforms for producers, industry associations, e-commerce platforms, and consumers. This will be an effective tool for strengthening risk self-management, industry self-discipline, and consumer rights protection and for improving the regulation efficiency of cross-regional food safety risks.

## Figures and Tables

**Table 1:** The main food production and consumption characteristics of sample areas in 2020.

Province	Meat Consumption *	Meat Output	Aquatic Product Consumption	Aquatic Product Output	Grain Consumption	Grain Output	Production and Consumption Characteristics
Shandong	18.6	35.9	15.7	81.5	124	537	Major food production regions
Jiangsu	25	17.6	19.5	57.8	122.1	440	Major food production regions
Anhui	24.1	35	14.6	38.1	148.3	659	Major food production regions
Fujian	24.6	26.1	26.4	200.2	124.4	121	Grain sales region
Zhejiang	26.3	8.9	25.9	91.1	137.3	94	Meat and grain sales region

* Data source: China Statistical Yearbook 2021. Unit: kg/person/year.

**Table 2:** Examples of valid and invalid information.

Name	Name of the Nominal Production Enterprise	Address of the Nominal Production Enterprise	Name of the Sampled Enterprise	Address of the Sampled Enterprise	Specification	Date of Production (Purchase or Quarantine)/Batch Number	Unqualified Items	Classification Result
Rice bar	Yantai Tianli Food Co., Ltd.	No.589 Huancheng Street, Muping District, Yantai City, Shandong Province	Chenjiali supermarket, Licang District	Room A-4, No.3 Yongping Road, Cangkou Sub-district, Licang District, Qingdao, Shandong Province	300 g/bag	16 April 2022	Peroxide number	Valid information
Red sausage	/	/	New Defu Roast Duck Shop, Wendeng District	No.3, Area B, Hongda Limin Market, No.8, Hengshan Road, Wendeng District, Weihai, Shandong Province	/	6 June 2022	Nitrite	Invalid information

**Table 3:** The proportion of cross-regional transferred unqualified products.

Food Categories	Number and Proportion of Detection Cross-Regional
Pastry	663 (15.6%)
Edible agricultural products	326 (7.67%)
Stir-fried products and nut products	269 (6.33%)
Convenience foods	226 (5.32%)
Seasonings	218 (5.13%)
Meat products	213 (5.01%)
Starch and starch products	210 (4.94%)
Quick-frozen food	210 (4.94%)
Liquor	199 (4.68%)

**Table 4:** High-risk categories in the national food safety inspection from 2016 to 2020.

Year	First Place	Second Place	Third Place	Fourth Place	Fifth Place
2020	Catering food	Vegetable products	Starch and starch products	Stir-fried products and nut products	Frozen products
2019	Vegetable products	Catering food	Convenience foods	Starch and starch products	Fruit products and aquatic products
2018	Vegetable products	Convenience foods	Catering food	Starch and starch products	Liquor
2017	Edible agricultural products	Special dietary food	Starch and starch products	Fruit products	Stir-fried products and nut products
2016	Fruit products	Aquatic products	Sugar	Starch and starch products	Special dietary food

**Table 5:** The food safety risk transfer network density.

Year	Binary Network *		Weighted Network	
	Density	Standard Deviation	Density	Standard Deviation
2016	0.0226	0.1486	0.0397	0.3202
2017	0.044	0.2051	0.0649	0.3571
2018	0.0731	0.2603	0.2087	1.3365
2019	0.068	0.2518	0.156	1.0359
2020	0.0822	0.2747	0.1589	0.7269

* The difference between binary network and weighted network is whether the connection is dual and whether the value of the connection between nodes is calculated, both of which can reflect the degree of connection between nodes. The weighted network can also reflect the average strength of connections but is susceptible to the strength of individual nodes.

**Table 6:** The centrality of the food safety risk transfer network.

Year	Centralization of the Network	Degree Centrality *		
		Average	Maximum	Minimum
2016	6.08%	4.58	31	0
2017	9.76%	7.69	44	0
2018	13.15%	24.62	356	1
2019	13.88%	18.15	285	1
2020	5.81%	17.51	49	2

* The centrality was based on the weighted network calculation, that is, not only whether there is food safety risk transfer between the two cities but also the number of risks.

**Table 7:** The substructure of the food safety risk transfer network in the five provinces from 2016 to 2020.

Year	Cliques	N-Clan Subgroups
2016	20	16
2017	56	48
2018	104	22
2019	106	27
2020	131	53
The 2016–2020 consolidated network	407	21

**Table 8:** Variables and descriptive statistics.

Variables	Variable Interpretation	Average	Variance
Centrality degree	The centrality of cities in the provincial food safety risk network	10.423	9.737
Territorial regulatory capacity	Whether a city is selected as a national food safety demonstration city	0.215	0.412
Intelligent supervision capability	Launch time of each city’s data open platform	0.298	0.458
Total value of agriculture	The logarithm value of total agricultural output	5.732	0.612
Total value of the food industry	The logarithm value of the total output value of agricultural and sideline product processing; food manufacturing; and the manufacturing of liquor, beverages, and refined tea	5.685	1.151
Total operating revenue of catering and accommodation	The logarithm value of the operating income of catering and accommodation above the quota	3.259	1.516
Per capita GDP	The logarithm value of the per capita national income	11.143	0.612
Engel coefficient	The ratio of the food expenditure to the total consumption expenditure, %	30.409	3.733
Urbanization rate	The proportion of the urban population in the total population of each city, %	0.63	0.097
Digital financial payment	Digital financial payment use index from the Digital Inclusive Finance Index	281.889	41.527

**Table 9:** Model test results.

Individual Effects Test	Hausman Test	Autocorrelation Test		Heteroscedasticity Test
		Cross-Section Autocorrelation	Sequence Autocorrelation	
F (64, 251) = 2.11	chi2(9) =195.92	Free’s test	F (1, 64) = 8.808	Prob > chi2 = 0.0000
Prob > F = 0.0000	Prob > chi2 = 0.0000	0747 (alpha = 0.05:0.686)	Prob > F = 0.0042	

**Table 10:** Empirical analysis result.

Variables	Coefficient	Driscoll–Kraay
		Standard Errors
Territorial regulatory capacity	−4.862 ***	0.803
Intelligent supervision capability	−0.755	0.678
Total value of agriculture	−13.219 *	5.712
Total value of the food industry	0.065	0.77
Total operating revenue of catering and accommodation	−0.727	0.505
Per capita GDP	4.395	2.945
Engel coefficient	0.287	0.413
Urbanization rate	22.550 **	7.972
Digital financial payment	0.064 *	0.029
Constant term	−0.521	30.244
F-test	29.31	
Prob > F	0.0027	

Note: *, ** and *** indicate significance at the 10%, 5% and 1% levels, respectively.

**Table 11:** Robust test.

Variables	Coefficient	Driscoll–Kraay
		Standard Errors
Territorial regulatory capacity	−3.028 **	1.030
Intelligent supervision capability	−1.052	0.753
Total value of agriculture	−10.920 **	3.748
Total value of the food industry	−0.968	1.590
Total operating revenue of catering and accommodation	1.233	0.735
Per capita GDP	1.162	3.628
Engel coefficient	0.084	0.719
Urbanization rate	1.915	16.356
Digital financial payment	0.100 *	0.042
Constant term	31.229	66.749
F-test	4.49	
Prob > F	0.0811	

Note: * and ** indicate significance at the 10% and 5% levels, respectively.

## Data Availability

The data presented in this study are available on request from the corresponding author.

## References

[1] Li T.P. (2020). Study on China’s Food Safety Situation Evaluation with Classification Random Sampling Theory. Issues Agric. Econ..

[2] Chai D., Meng T., Zhang D. (2022). Influence of food safety concerns and satisfaction with government regulation on organic food consumption of Chinese urban residents. Foods.

[3] Kendall H., Kaptan G., Stewart G., Grainger M., Kuznesof S., Naughton P., Clark B., Hubbard C., Raley M., Marvin H.J.P. (2018). Drivers of existing and emerging food safety risks: Expert opinion regarding multiple impacts. Food Control.

[4] Unnevehr L.J. (2022). Addressing food safety challenges in rapidly developing food systems. Agric. Econ..

[5] Hu Y.L. (2019). Market Regulation in China: Starting Point, Key Issues and Research Subjects. China Public Adm..

[6] Song B., Yan W., Zhang T. (2019). Cross-border e-commerce commodity risk assessment using text mining and fuzzy rule-based reasoning. Adv. Eng. Inform..

[7] Motarjemi Y., Käferstein F. (1999). Food safety, Hazard Analysis and Critical Control Point and the increase in foodborne diseases: A paradox?. Food Control.

[8] Devaney L. (2016). Good governance? Perceptions of accountability, transparency and effectiveness in Irish food risk governance. Food Policy.

[9] Al-Busaidi M.A., Jukes D.J., Bose S. (2017). Hazard analysis and critical control point (HACCP) in seafood processing: An analysis of its application and use in regulation in the Sultanate of Oman. Food Control.

[10] Wu Y., Chen J. (2018). Food safety monitoring and surveillance in China: Past, present and future. Food Control.

[11] Liu C., Zhang H., An Y.F. (2011). Study on Weaknesses, Root Causes and Key Issues of China’s Food Quality Safety: Based on the Empirical Analysis of 1, 460 Food Quality Safety Cases. Issues Agric. Econ..

[12] Li S.G., Chen L.L., Chen B. (2014). The Analysis of Food Safety Incidents Exposed by the Media from 2004 to 2012 in China. J. Chin. Inst. Food. Sci. Technol..

[13] Wen X.W., Liu M.L. (2012). Cause, Dilemma and Supervision of Food Safety from the Year 2002 to 2011. Reform.

[14] Zhu D., Hong X.J. (2014). The Study of Food Safety Risk Assessment and Risk Characteristics in China during 2006–2012. Chin. Rural Surv..

[15] Zhang H.X. (2021). Identification and Distribution Characteristics of Food Safety Risk Factors-An Empirical Analysis Based on 9314 Food Safety Incidents. Contemp. Econ. Manag..

[16] Tao Q.H., Yang X., Song Y.J., Jin J.Y. (2021). Analysis of Food Safety Sampling Data in China from 2017 to 2019. Sci. Technol. Food. Ind..

[17] Beni L.H., Villeneuve S., LeBlanc D.I., Côté K., Fazil A., Otten A., McKellar R., Delaquis P. (2012). Spatio-temporal assessment of food safety risks in Canadian food distribution systems using GIS. Spat. Spatio-Temporal Epidemiol..

[18] Grace D. (2015). Food safety in low and middle income countries. Int. J. Environ. Res. Public Health.

[19] Wang J., Chen T. (2016). The spread model of food safety risk under the supply-demand disturbance. Springer Plus.

[20] Jin C.Y., Levi R., Liang Q., Renegar R., Springs S., Zhou J.H., Zhou W.H. (2021). Testing at the source: Analytics-enabled risk-based sampling of food supply chains in China. Manag. Sci..

[21] Cabo M.L., Romalde J.L., Simal-Gandara J., Martínez A.G., Fernández J.G., Costas M.B., Hierro S.P., Ortega A.P., Manaia C.M., Silva J.A. (2020). Identification of emerging hazards in mussels by the Galician emerging food safety risks network (RISEGAL). A first approach. Foods.

[22] Li Q.G., Li Y.Q., Niu L.Y., Wu L.H., Hong W. (2016). Spatial Distribution and Changing Trend of Food Safety Incidents in China. Econ. Geogr..

[23] Zhang H.B. (2017). Research on Food Safety about its Risk Communication and Collaborative Governance-Taking Exposure Events from 2007–2016 as Research Object. J. Intell..

[24] Yan Z., Liu Q., Wu S.S. (2020). Risk Evolution and Spatial Transformation of Agricultural Product Safety and Quality Issues-Evidence from Media Coverages. J. Agric. Econ..

[25] Nie W.J. (2022). Spatial Diffusion and Driving Mechanism of Food Safety Risk in China from the Perspective of Regulatory Intensity. Mod. Econ. Res..

[26] Nayak R., Waterson P. (2019). Global food safety as a complex adaptive system: Key concepts and future prospects. Trends Food Sci. Technol..

[27] Ercsey-Ravasz M., Toroczkai Z., Lakner Z., Baranyi J. (2012). Complexity of the international agro-food trade network and its impact on food safety. PLoS ONE.

[28] Natale F., Giovannini A., Savini L., Palma D., Possenti D., Fiore G., Calistri P. (2009). Network analysis of Italian cattle trade patterns and evaluation of risks for potential disease spread. Rev. Vet. Med..

[29] Verhaelen K., Bauer A., Günther F., Müller B., Nist M., Celik B., Weidner C., Küchenhoff H., Wallner P. (2018). Anticipation of food safety and fraud issues: ISAR-A new screening tool to monitor food prices and commodity flows. Food Control.

[30] Granovetter M.S. (1985). Economic Action and Social Structure: The Problem of Embeddedness. Am. J. Sociol..

[31] Liu H.J., Liu C.M., Yang Q. (2015). Spatial spillover and the source of environment pollution-Empirical study on the perspective of network analysis. Economist.

[32] He C.F., Wu W.J. (2021). China’s Food Export Network and its Evolution. Hum. Geogr..

[33] Zhang H.F., Huang L., Sun C.Y. (2021). Government Intervention and Development Efficiency of Food Industry- From the Perspective of Local Government Competition. Rev. Econ. Manag..

[34] Liu P., Ma L. (2016). Food scandals, media exposure, and citizens’ safety concerns: A multilevel analysis across Chinese cities. Food Policy.

[35] Iftekhar A., Cui X. (2021). Blockchain-based traceability system that ensures food safety measures to protect consumer safety and COVID-19 free supply chains. Foods.

[36] Ni G.H., Niu X.Y., Liu Q. (2019). Can “Keeping Secret” for Food Safety Incidents Protect the Food Industry? Based on the Empirical Analysis of 2896 Food Safety Incidents. J. Agric. Econ..

[37] Xu G.C., Li W.R. (2021). Influencing Factors and Governance Paths of Food Safety Accidents QCA Analysis Based on REASON Model. J. Manag..

[38] Liu P., Zhong X. (2021). Is Smart Supervision Really Smart? --A Case Study on the Reform of Food Safety Supervision Conducted by Local Governments. J. Guangxi Norm. Uni. (Philos. Soc. Sci.).

[39] Hu Y.L. (2020). Modernization of Food Safety Governance: Starting Point, Framework and Task Force. J. Macro-Qual. Res..

[40] Zhan C.Y. (2019). The Evolutionary Logic of China’s Food Safety Regulatory System Reform and the Problem to Be Solved. Nanjing. J. Soc. Sci..

[41] Jiang X.J., Huang Y.X. (2021). Market Order, Market Supervision and Platform Governance in the Digital Age. Econ. Res. J..

[42] Ye L., Wang Y.Q. (2019). Policy Process and Innovation Mechanism of Grassroots Smart Regulation-Case Studies of District Administration for Market Regulation in Eastern Coastal Cities. China Public Adm..

[43] Liu P., Li W.T. (2018). Food Safety Regulation of Online Meal Ordering: A Study from the Perspective of Smart Regulation. J. Cent. China Norm. Uni. (Hum. Soc. Sci.).

[44] Li T., Feng H.X. (2022). Digital Governance: Its Multi-dimensional Perspective, Scientific Connotation and Basic Elements. Soc. Sci. Digit..

[45] Zhang M.H., Wen J.F., Liu Z.J. (2017). Industry self-discipline, social supervision and vertical collaboration: Research on food safety behavior based on the perspective of social co-governance. Ind. Econ. Res..

[46] Wang X.Q. (2000). China’s Food Industry: Growth, Structure and Performance. Chin. Rural Econ..

[47] Zhang W. (2022). Research on the Relationship between Food Safety Regulation, Food Industry Technology Innovation and Food Quality Improvement. Econ. Rev. J..

[48] Zhou Y.H., Wang S.G., Yan B.J. (2022). The Structure, Evolution and Prospect of Food System in China. Issues Agric. Econ..

[49] Zhang H.F., Jiang Q., Lv J. (2019). Economic Growth and Food Safety: FKC Hypothesis Test and Policy Implications. Econ. Res. J..

[50] Yin S.J., Wang J.B., Wu H.L. (2021). Does the Food Safety Risk Kuznets Curve Exist?—Evidence from the Provincial Food Safety Incidents Reported on the Internet. China Bus. Mark..

[51] Matuschke I. (2009). Rapid Urbanization and Food Security: Using Food Density Maps to Identify Future Food Security Hot Spots.

[52] Hon-Ming L., Justin R., Ming-Chiu F., Xu L., Sun S.S.-M. (2013). Food supply and food safety issues in China. Lancet.

[53] Du S.C. (2019). An Alternative Mode of Trust: Construction of Trust in Online Consumption. China Stud..

